# Welche Bedeutung hat die Bindehaut als möglicher Übertragungsweg für eine SARS-CoV-2-Infektion?

**DOI:** 10.1007/s00347-020-01150-1

**Published:** 2020-06-22

**Authors:** Clemens Lange, Julian Wolf, Claudia Auw-Haedrich, Anja Schlecht, Stefaniya Boneva, Thabo Lapp, Hansjürgen Agostini, Gottfried Martin, Thomas Reinhard, Günther Schlunck

**Affiliations:** 1 Klinik für Augenheilkunde, Universitätsklinikum Freiburg, Killianstr. 5, 79106 Freiburg, Deutschland; 2 Medizinische Fakultät, Universität Freiburg, Breisacher Str. 153, 79110 Freiburg, Deutschland

**Keywords:** SARS-CoV‑2, COVID-19, ACE2, TMPRSS2, Bindehaut, SARS-CoV‑2, COVID-19, ACE2, TMPRSS2, Conjunctiva

## Abstract

Aktuelle Studien haben bei ca. 1 % aller COVID-19-Patienten eine Bindehautentzündung beschrieben und spekuliert, dass SARS-CoV‑2 über die Bindehaut übertragen werden kann. In der vorliegenden Arbeit rekapitulieren wir die molekularen Mechanismen des Eintritts von SARS-CoV‑2 in die Wirtszelle und diskutieren die aktuelle Studienlage zu einer möglichen konjunktivalen Transmission. Derzeit geht man davon aus, dass SARS-CoV‑2 das membrangebundene Angiotensin-konvertierende Enzym 2 (ACE2) sowie die Membran-gebundene Serinprotease TMPRSS2 benötigt, um in die Wirtszelle einzudringen. Aktuelle Studien weisen darauf hin, dass COVID-19-Patienten nur sehr selten Virus-RNA im Tränenfilm und Bindehautabstrichen aufweisen und dass *ACE2* und *TMPRSS2* in der Bindehaut nur in sehr geringen Mengen gebildet werden, was eine konjunktivale Infektion durch SARS-CoV‑2 über diese Mediatoren wenig wahrscheinlich macht. Dennoch halten wir die derzeitige Studienlage für zu begrenzt, um eine abschließende Aussage treffen zu können, und empfehlen konsequente und adäquate Schutzmaßnahmen für medizinisches Personal, das in engem Kontakt mit verdächtigen und bestätigten COVID-19-Patienten steht.

## Abstract (en)

Recent studies have described conjunctivitis in approximately 1% of COVID-19 patients and speculated that SARS-CoV‑2 can be transmitted via the conjunctiva. In this article we recapitulate the molecular mechanisms of host cell entry of SARS-CoV‑2 and discuss the current evidence for a potential conjunctival transmission of SARS-CoV‑2. The current body of evidence indicates that SARS-CoV‑2 requires the membrane-bound angiotensin-converting enzyme 2 (ACE2) and the membrane-bound serine protease TMPRSS2 to enter cells. Recent studies suggest that COVID-19 patients rarely exhibit viral RNA in tear film and conjunctival smears and that, *ACE2* and *TMPRSS2* are only expressed in very small amounts in the conjunctiva, making conjunctival infection with SARS-CoV‑2 via these mediators unlikely. Nevertheless, we consider the current evidence to be still too limited to provide a conclusive statement and recommend appropriate protective measures for healthcare personnel who are in close contact with suspected and confirmed COVID-19 patients.

Der Ausbruch von COVID-19 wurde von der WHO zu einer gesundheitlichen Notlage internationaler Tragweite erklärt. COVID-19 wird durch das schwere akute respiratorische Syndrom Coronavirus‑2 (SARS-CoV-2) übertragen und ist mit Symptomen wie Fieber, Husten, Geschmacksstörungen und Müdigkeit sowie einer schweren Lungenentzündung vergesellschaftet [19]. SARS-CoV‑2 ist hochgradig infektiös und wird hauptsächlich durch das Einatmen von Tröpfchen oder Aerosolen, die von einer infizierten Person freigesetzt werden, und möglicherweise auch über den fäkooralen Weg übertragen [6]. Eine potenzielle konjunktivale Übertragung von SARS-CoV‑2 ist nicht abschließend geklärt und würde erhebliche Auswirkungen auf die öffentliche Gesundheit haben. So postulieren einzelne Studien, dass SARS-CoV‑2 über die Schleimhäute einschließlich der Bindehaut übertragen werde [2, 3] und dass alle Augenärzte einem erhöhten Risiko ausgesetzt seien und somit bei der Untersuchung von Verdachtsfällen Schutzbrillen tragen sollten [14].

## Infektionsweg und Replikation von SARS-CoV-2

**Figure 1:**
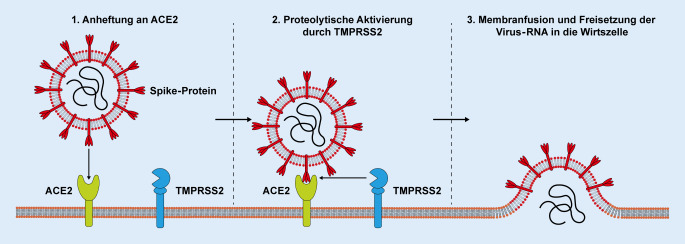

Ähnlich wie SARS-CoV nutzt SARS-CoV‑2 das membrangebundene Angiotensin-konvertierende Enzym 2 (ACE2) sowie die membrangebundene Serinprotease TMPRSS2, um in die Wirtszelle einzudringen [7]. SARS-CoV‑2 bindet dabei mit seinem Spike(S)-Glykoprotein an ACE2 und kann durch TMPRSS2-vermittelte proteolytische Aktivierung des Spike-Proteins mit der Wirtsmembran fusionieren (Abb. 1; [7, 16]). Nach der Membranfusion wird die RNA des Virus freigesetzt, repliziert und an den Ribosomen der Wirtszelle in virusspezifische Proteine umgeschrieben. Es wird angenommen, dass SARS-CoV‑2 ähnlich wie SARS-CoV in das endoplasmatische Retikulum aufgenommen wird und die Wirtszelle durch Exozytose verlassen kann. Seit dem Ausbruch von COVID-19 hat eine Reihe von Studien die Expression von ACE2 in verschiedenen menschlichen Geweben untersucht und neben einer Expression in Lungengewebe auch eine deutliche Expression des Rezeptors in Magen‑, Kolon‑, Leber- und Nierengewebe nachgewiesen [15, 23]. Dies verdeutlicht die Anfälligkeit verschiedenster Gewebe für eine SARS-CoV-2-Infektion und erklärt die klinische Beobachtung einer möglichen Beteiligung mehrerer Organe im Rahmen einer SARS-CoV-2-Infektion [19]. Es ist derzeit nicht eindeutig geklärt, ob Zellen der Augenoberfläche ACE2 oder TMPRSS2 exprimieren und damit für eine SARS-CoV-2-Infektion anfällig sind.

## Möglichkeiten der Infektion mit SARS-CoV-2 über die Augenoberfläche

Zur Möglichkeit einer Virusübertragung über die Augenoberfläche ergeben sich 3 wesentliche Fragen.

1. *Kann SARS-CoV-2 die Bindehaut infizieren und sich dort replizieren?*

**Figure 2:**
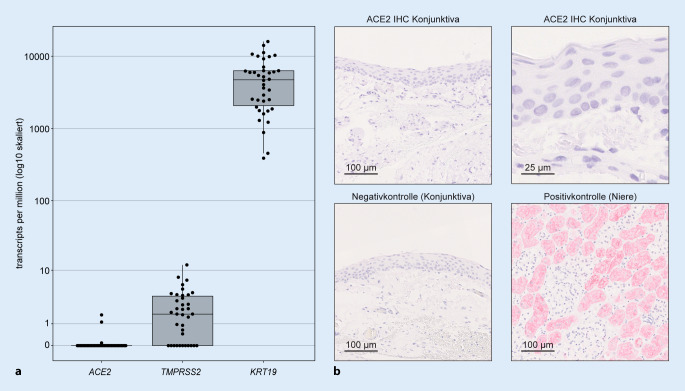

Aktuelle Studien beschreiben bei ca. 7 % aller COVID-19-Patienten subjektive okuläre Symptome [22] und bei ca. 1 % Zeichen einer Konjunktivitis [5]. Diesen Beobachtungen liegt jedoch nur selten eine ophthalmologische Untersuchung zugrunde, und die Einschlusskriterien sind uneinheitlich [10]. Zudem wurden in den genannten Studien keine adäquaten Kontrollkohorten untersucht, und es ist unklar, ob die Symptome durch SARS-CoV‑2 bedingt wurden oder ob es sich um SARS-CoV-2-unabhängige Epiphänomene, z. B. im Zuge der intensivmedizinischen Behandlung, handelt. Des Weiteren ist es umstritten, ob Zellen der Augenoberfläche, wie z. B. konjunktivale Epithelien, ACE2 oder TMPRSS2 exprimieren und damit für eine SARS-CoV-2-Infektion anfällig sind. Angesichts der unklaren Datenlage haben wir kürzlich das Expressionsniveau von *ACE2* und *TMPRSS2* in 38 gesunden und erkrankten Bindehautproben untersucht [11]. Dazu wurde RNA aus Formalin-fixierten und in Paraffin eingebetteten Bindehautproben wie zuvor beschrieben isoliert, sequenziert [12, 17], und anschließend die Sequenzierungsdaten bioinformatisch ausgewertet [1]. Während die Bindehautproben eine erhebliche mRNA-Expression des Epithelmarkers *Keratin 19* aufwiesen, zeigten gesunde und erkrankte Bindehautproben keine relevante Expression des SARS-CoV-2-Rezeptors *ACE2* (Abb. 2a). Im Einklang mit der kaum nachweisbaren Expression von *ACE2* auf transkriptioneller Ebene fanden wir zudem eine vernachlässigbare ACE2-Immunreaktivität in 8 gesunden Bindehautproben [11], was auf das Fehlen einer relevanten ACE2-Proteinexpression in der Bindehaut hinweist (Abb. 2b). Darüber hinaus zeigen unsere Daten, dass auch die Serinprotease *TMPRSS2* in Bindehautgewebe nicht wesentlich transkribiert wird (Abb. 2a). Diese Ergebnisse sprechen gegen einen ACE2-vermittelten konjunktivalen Infektionsweg von SARS-CoV‑2 und sind im Einklang mit histologischen Untersuchungen von an COVID-19 verstorbenen Patienten, die keine relevante Bindehautentzündung nachweisen konnten [13]. Damit scheint sich SARS-CoV‑2 von anderen Viren, wie z. B. Hepatitis-C-Viren, zu unterscheiden, deren konjunktivale Infektion und Übertragung beschrieben sind [8]. Auch wenn bislang entsprechende Hinweise fehlen, bleibt zu klären, ob individuelle Faktoren wie Hypoxie oder Rauchen eine Expression von ACE2 in der Bindehaut auslösen können [21]. In einem ex-vivo-Modell berichtet eine aktuelle Untersuchung über die Möglichkeit der Infektion von Zellen v. a. des Bindehautstromas durch SARS-CoV‑2 [9]. Es bleibt aber unklar, inwiefern sich diese Beobachtung auf die Situation in vivo übertragen lässt. Zudem scheint eine aggressivere Vorbehandlung von histologischen Bindehautpäparaten eine ACE2 Immunreaktivität des Bindehautepithels zu ermöglichen, deren klinische Relevanz jedoch unklar ist [4]. Weitere Untersuchungen z. B. an Obduktionsmaterial von an COVID-19 Verstorbenen sind notwendig, um Aufschluss über die tatsächliche Infektiosität und mögliche Orte der Virusreplikation zu erhalten.

2. *Können Gesunde über den Tränenfilm infiziert werden?*

Zur Infektion Gesunder mit SARS-CoV‑2 über den Tränenfilm liegen derzeit keine Daten vor. Während einige Daten (s. oben) gegen eine Infektion der Bindehaut durch SARS-CoV‑2 sprechen, könnten im Tränenfilm befindliche Viren grundsätzlich über die ableitenden Tränenwege Zugang zur Nasenschleimhaut und den Atemwegen erhalten und so eine Infektion respiratorischer Epithelien auslösen. Es ist derzeit unklar, inwiefern durch Reiben der Augen mit kontaminierten Händen eine Ansteckung über den Tränenfilm möglich ist. Über die Bedeutung der Virusaufnahme über die Tränenwege im Vergleich zur direkten Aufnahme von virushaltigen Aerosolen über die Atemwege kann auch nur spekuliert werden. Aufgrund des schützenden Lidschlags und der kleineren Oberflächen dürfte ein rein okulärer Infektionsweg für SARS-CoV‑2 eine untergeordnete Rolle spielen. Allerdings erscheint ein Schutz der Augen bei engem Kontakt oder hohem Expositionsrisiko, wie z. B. der trachealen In- oder Extubation von an COVID-19 Erkrankten, dringend geboten.

3. *Ist der Tränenfilm Erkrankter infektiös?*

Zwei kürzlich veröffentlichte Studien konnten nur bei einem geringen Anteil von COVID-19-Patienten SARS-CoV-2-RNA in Bindehautabstrichen nachweisen [20, 22]. Zhou et al. analysierten die Bindehautabstriche von 67 bestätigten oder vermuteten COVID-19-Fällen und berichteten, dass nur bei 1 Patienten ein positives und bei 2 Patienten ein wahrscheinlich positives PCR-Ergebnis vorlag. Keiner der 3 Patienten wies eine Bindehautentzündung auf [22]. In ähnlicher Weise untersuchten Xia et al. insgesamt 30 Patienten mit bestätigtem SARS-CoV-2-Nachweis in Sputumproben und berichteten, dass nur bei einem dieser Patienten SARS-CoV-2-RNA auch im Bindehautabstrich nachgewiesen werden konnte. Bei diesem Patienten lagen auch Anzeichen einer Bindehautentzündung vor [20]. In einer weiteren Studie untersuchten Seah et al. 17 COVID-19-Patienten mit positivem nasopharyngealem Abstrich in stationärer Behandlung. Auch in mehrfacher Testung gelang bei keinem der Patienten eine Virusisolation oder der Nachweis von SARS-CoV‑2 im Tränenfilm [18]. Diese Daten sprechen dafür, dass selbst bei Patienten mit florider COVID-19-Erkrankung der Tränenfilm nur sehr selten Virus-RNA enthält. Der Nachweis von viraler RNA kann nicht gleichgesetzt werden mit dem Vorhandensein infektiöser Viruspartikel. Somit scheint das Risiko der Ansteckung mit SARS-CoV‑2 durch Tränenflüssigkeit von infizierten Patienten gering zu sein.

## Schlussfolgerung

Die aktuelle Studienlage lässt aufgrund der geringen Anzahl an untersuchten COVID-19-Patienten keine abschließende Aussage über eine mögliche SARS-CoV-2-Infektion der Bindehaut zu. Da COVID-19-Patienten jedoch nur selten klinische Anzeichen für eine Bindehautinfektion aufweisen und SARS-CoV-2-RNA nur sporadisch in der Tränenflüssigkeit nachgewiesen wurde, erscheint eine konjunktivale SARS-CoV-2-Infektion unwahrscheinlich. Diese klinischen Beobachtungen werden durch grundlagenwissenschaftliche Arbeiten gestützt, die eine geringe Expression von *ACE2* und *TMPRSS2 *beschreiben. Dies macht eine konjunktivale SARS-CoV-2-Transmission über diese Mediatoren unwahrscheinlich – schließt andere Infektionswege über bislang unbekannte Rezeptoren aber nicht aus. Zudem könnte die Inokulation von SARS-CoV‑2 durch Tränen erfolgen, die das Virus über das nasolakrimale Drainagesystem in den Nasen-Rachen-Raum transportieren und dort Zellen infizieren. Weitere Untersuchungen, z. B. an Obduktionsmaterial von an COVID-19 Verstorbenen sind notwendig, um Aufschluss über die tatsächliche Infektiosität und mögliche Orte der Virusreplikation zu erhalten. Bis diese Eventualitäten mit Sicherheit ausgeschlossen sind, sollten wirksame Schutzmaßnahmen für Ärzte mit engem Kontakt zu COVID-19 angewandt werden, die Mund und Nase und ggf. die Augen schützen. Für die augenärztliche Praxis dürfte von Aerosolen aus den Atemwegen und dem nahen Kontakt zum Patienten bei bestimmten augenärztlichen Untersuchungen ein höheres Infektionsrisiko ausgehen als von Tränenfilm und Augenoberfläche der Patienten.
